# Impact of precursor dosing on the surface passivation of AZO/AlO_*x*_ stacks formed using atomic layer deposition†

**DOI:** 10.1039/d4ya00552j

**Published:** 2025-02-03

**Authors:** Yan Wang, Theodore D. C. Hobson, Jack E. N. Swallow, Shona McNab, John O’Sullivan, Anastasia H. Soeriyadi, Xinya Niu, Rebekah C. Fraser, Akash Dasgupta, Soumyajit Maitra, Pietro P. Altermatt, Robert S. Weatherup, Matthew Wright, Ruy S. Bonilla

**Affiliations:** a Department of Materials, University of Oxford Oxford OX1 3PH UK sebastian.bonilla@materials.ox.ac.uk; b School of Photovoltaic and Renewable Energy Engineering, University of New South Wales Sydney 2052 Australia; c Department of Physics, University of Oxford Oxford OX1 3PU UK

## Abstract

High-efficiency solar cell architectures, including silicon heterojunction (SHJ) and perovskite/silicon tandems, rely heavily on the unique properties of transparent conducting oxides (TCOs). The push towards terawatt-scale PV manufacturing means it is increasingly desirable to develop indium-free TCOs to facilitate the upscaled manufacturing of high-efficiency cell designs. Aluminium-doped ZnO (AZO) deposited by atomic layer deposition (ALD) has emerged as a promising candidate due to its combination of optical transparency and electrical conductivity. In addition, AZO has also been shown to passivate the c-Si surface. The ability for one material to provide all three properties without requiring any indium is advantageous in single junction and tandem solar devices. Herein, we demonstrate exceptional silicon surface passivation using AZO/AlO_*x*_ stacks deposited with ALD, with a *J*_0_ < 1 fA cm^−2^ and corresponding implied open circuit voltage (iV_OC_) of 740 mV. We provide a comprehensive analysis of the role of ALD precursor dosing to achieve optimised performance. A broad range of characterisation approaches were used to probe the structural, compositional, and chemical properties of AZO films. These indicated that the passivation properties are governed by a delicate interplay between the Zn and Al concentrations in the film, highlighting the importance of precise process control. Optical modelling in a single junction SHJ architecture indicates these AZO films are close in performance to high-mobility indium-containing TCOs. The insights provided by this work may help to further the case of indium-free TCOs, which is critical for upscaled production of high-efficiency solar cells.

## Abstract 2

Aluminium-doped ZnO deposited by ALD provides transparency, conductivity, and c-Si passivation for high-efficiency solar cells, reducing the need for indium-based TCOs. Precise Zn/Al control enables passivation with *J*_0_ < 1 fA cm^−2^ and 740 mV iV_OC_.

## Graphical abstract

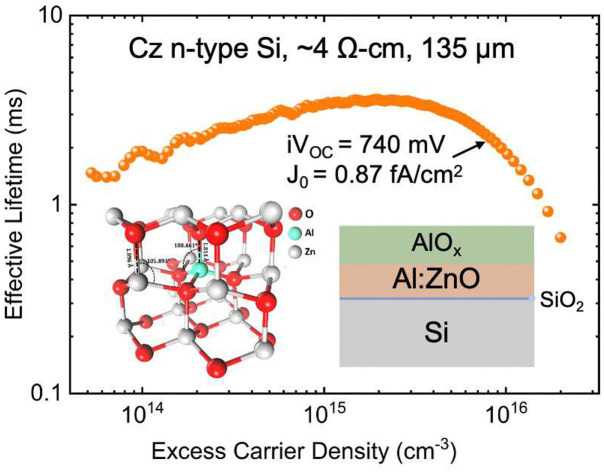

## Introduction

Achieving net-zero carbon emissions requires deploying terawatts (TW) of renewable energy.^1^ While photovoltaic (PV) solar cells currently contribute only 5.5% to global electricity, they have enormous potential to deliver over 50% of the world's electricity needs in the next two decades.^2^ For this growth to materialise, the power output in all future solar farms must be maximised.^3^ This requires methods to minimise losses and maximise solar cell power conversion efficiency.^4^ Transparent conducting oxides (TCO) are increasingly relevant for advancing high-performance solar cell structures and scalable industrial production.^5^ Si heterojunction (SHJ) solar cells, on which recent world records have been demonstrated,^6–9^ require a TCO in their device structure. The emergence of high-efficiency perovskite–silicon tandem architectures, in which two semiconductor absorbers are used to harvest more energy from sunlight, will make TCOs even more critical as the connection of two semiconductors is achieved *via* transparent conducting interlayers.^10^

Most commonly used TCOs in the solar industry contain indium. Indium-based TCOs, such as tin-doped indium oxide (ITO) and tungsten-doped indium oxide (IWO), tend to have high free carrier mobility, allowing for simultaneous high transparency in the visible range and sufficient conductivity for PV devices.^5^ However, indium is scarce, and using it in TCOs limits TW-scale deployment of PV.^11^ Hence, PV cells dependent on indium cannot contribute significantly to climate change mitigation. Currently, ongoing research aims to find alternative TCO materials that are earth-abundant while providing the necessary properties for high-efficiency PV devices. One of the promising indium-free TCO alternatives is doped zinc oxide (ZnO).^12^ Doping ZnO with aluminium (AZO) provides sufficient conductivity for TCO functionality in solar cells. Researchers have also used a variety of deposition techniques to fabricate ZnO, including spray pyrolysis,^13^ chemical vapor deposition (CVD),^14^ pulsed laser deposition (PLD),^15,16^ atomic layer deposition (ALD),^17–19^ sputtering,^20,21^ and sol–gel processing.^22^ Among these, ALD has recently demonstrated several crucial advantages, such as atomic-level thickness control, excellent spatial uniformity, industrial scalability, and doping tailoring.^23^ Furthermore, ALD-ZnO materials also show promising properties for device integration, including interface passivation, good optical coupling, and processing compatibility.^24^

Besides transparency and conductivity, PV devices require outstanding surface passivation. Minimising interface recombination losses remains one of the major hurdles in the pathway to ultra-high-efficiency solar cells. We use two parameters to quantify passivation: implied open circuit voltage (iV_OC_) and saturation current density (*J*_0_). iV_OC_ represents the maximum open-circuit voltage (*V*_OC_) possible in a solar cell, assuming unity ideality factor in the diode equation. It is calculated from quasi-steady-state (QSS) or transient photoconductance decay (PCD), or photoluminescence (PL) measurements of the solar absorber before charge-extracting electrodes are applied. The QSS PCD measurement is well established^25^ and uses the recorded average excess carrier concentration (Δ*n*) to infer the voltage performance of the cell under 1 sun (AM1.5G) standard illumination conditions. A high iV_OC_ indicates good passivation quality and is closely correlated with higher solar cell efficiency. For instance, silicon solar cells with iV_OC_ values reaching 730–750 mV correspond to high open-circuit voltage in complete cells, leading to efficiencies above 25%.^26–28^

The *J*_0_ current density characterises the total recombination losses, both in the bulk, the surfaces, and metal contacts of the solar cell. Lower *J*_0_ values indicate reduced recombination and, thus better passivation quality. For instance, in well-passivated silicon solar cells, *J*_0_ values below 10 fA cm^−2^ are required for efficiencies to exceed 25%.^29,30^

Traditionally, the purpose of a TCO is to achieve high transmission of light into the absorber while having sufficient conductivity to extract photo-generated electrons towards the metal contacts with minimal resistive losses. However, TCOs may also function as a passivation layer, for example, on the crystalline silicon (c-Si) surface^31,32^ or the charge transport layer interface in a perovskite cell.^33,34^ Recent reports have demonstrated that excellent passivation of a c-Si surface is possible using SiO_*x*_/AZO/AlO_*x*_. Macco *et al.* showed significant advancements in the use of AZO as a transparent, passivating and conductive layer for solar cells, demonstrating the potential for iV_OC_ as high as 728 mV.^32^ This improvement was largely attributed to ALD deposition. The use of supercycle growth leads to a layered structure of dopants embedded in the host ZnO matrix, rather than being isotropically distributed.^35^ This non-uniform distribution can influence field-effect passivation by affecting the distribution of charge carriers at the surface, which impacts recombination. Therefore, understanding how ALD preparation conditions affect electrical conductivity and passivation behaviour is crucial for exploiting all functionalities in AZO films for solar cell applications.

Previous studies have demonstrated that excellent carrier transport properties were achievable using dimethylaluminum isopropoxide (DMAI) as the Al precursor in AZO films on Si passivated with a-Si.^36^ This success was primarily attributed to the steric hindrance of the larger isopropoxyl ligand (O^i^Pr, approximately 4.9 Å in size).^37^ The Al atoms from DMAI are spaced farther apart during deposition compared to smaller precursors like trimethylaluminum (TMA). The increased spacing can prevent unwanted clustering of Al atoms on the ZnO surface, thus decreasing inactive regions where the Al atoms cannot effectively substitute into the ZnO lattice so that a higher doping efficiency can be achieved. Therefore, the DMAI precursor results in enhanced charge carrier mobility.^38^ In comparison, the more common TMA precursor was shown to be ineffective at shielding surface area and sterically hindering further reactions between adjacent –OH surface groups and incoming precursor molecules.^37^ In TMA, the methyl ligand CH_3_ is approximately 2.6 Å in size. Lower doping efficiency of Al from TMA compared with DMAI would mean lower carrier densities from ineffective Al atoms. These inactive dopants can act as scattering centres, leading to low mobility.

This work demonstrates that a combination of favourable transparent conducting electrode (TCE) properties and improved surface passivation is possible using TMA as the Al precursor for AZO, provided the dosing conditions are carefully monitored and optimised. We achieved an optimised ALD recipe yielding outstanding surface passivation, with the lowest reported *J*_0_ of 0.87 fA cm^−2^ corresponding to iV_OC_ of 740 mV. Our optimised AZO achieves a stringent combination of functions: lateral conductivity across a nanolayer thinner than 40 nm, effective chemical and field-effect passivation of the Si surface, and low optical losses *via* well-matched refractive index and low absorption.

We present the impact that precursor dosing pressures have on the passivation properties, and we use such pressures as the tuning parameter to achieve the best passivation performance. We also investigated the interface properties and AZO/AlO_*x*_ passivation mechanisms to establish the contributions of interface chemistry and field effect to optimised performance. This work highlights the impact and insights into how ALD precursors affect AZO film deposition and influence surface passivation and optoelectronic properties. Our work paves the way for future mass production of solar cells using earth-abundant TCO materials that provide high transparency, conductivity, and passivation.

## Precursor dosing for optimal surface passivation

The use of AZO as a transparent and passivating TCO on n-type Si has been previously realised *via* DMAI, leading to an iV_OC_ of 728 mV and *J*_0_ of >10 fA cm^−2^.^32^ Here, we develop and study an ALD-AZO recipe that instead uses TMA as the precursor for Al doping of ZnO. We focus our development on the optimisation of the passivation properties of AZO, demonstrating that it is possible to improve iV_OC_ to 740 mV with the use of TMA Al doping, while still achieving favourable TCE properties. Our best minority carrier lifetime result also corresponds to one of the lowest reported *J*_0_ values in the literature, 0.87 fA cm^−2^.

**Fig. 1:**
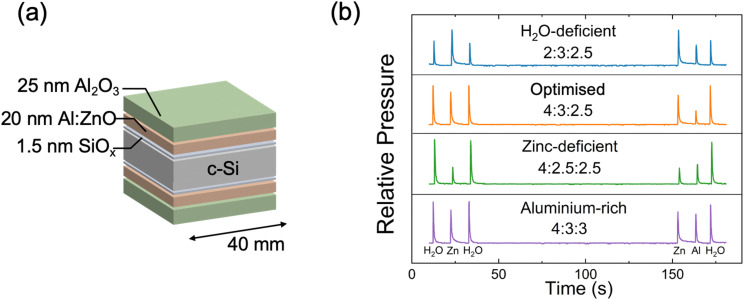
(a) Schematic diagram of the sample for passivation measurements; (b) pressure ratio profiles of different precursor purges in ALD. Note: the pressure profiles are offset to allow for comparison.

Symmetrical SiO_*x*_/AZO/AlO_*x*_ passivation stacks were fabricated on n-type Si, as illustrated in Fig. 1a. While the AZO layer can be exploited as a transparent conductor, the central role we explore is the recombination losses at the interface. The top AlO_*x*_ served as a capping layer for hydrogen passivation purposes.^32^ The SiO_*x*_ was chemically grown by RCA2, while the AZO and AlO_*x*_ layers were grown bifacially on the c-Si wafer, ensuring uniform and equivalent passivation characteristics on both sides of the Si specimen. Additionally, the AZO and AlO_*x*_ are grown sequentially inside the same thermal ALD system, demonstrating high process compatibility.

In our AZO process development, we evaluated the surface passivation of sample batches deposited over the span of a year. We found significant discrepancies in the passivation across different batches, which prompted a review of the synthesis conditions. We examined the gas dosing and found that the precursor gas ratios during ALD deposition were the primary descriptor correlating to the changes in AZO passivation properties. Careful monitoring and reproducibility of precursor dosing emerged as the key to achieving high surface passivation. To exemplify our yearlong findings, this work describes four recipes designed to investigate how deposition conditions influence surface passivation on n-type Si.

In this work, we vary the flow of gas precursors H_2_O, diethylzinc (DEZ) and TMA, such that four distinct recipes with varying gas partial pressures are achieved. The gas pressure profiles for the four recipes are shown in Fig. 1b. Modulating the precursor gas flow allows for the formation of H_2_O-deficient (blue curve), Zn-deficient (green curve), and Al-rich (purple curve) AZO films. The recipe enabling the highest passivation AZO is labelled “optimised”, shown as the orange curve. The absolute peak precursor pressures for each of the recipes are shown in Table 1, along with the H_2_O : DEZ : TMA ratios.

**Table 1:** The approximate pressure peak for each precursor and their ratios in the four different ALD pressure profiles

Group	H_2_O pressure (mTorr)	DEZ pressure (mTorr)	TMA pressure (mTorr)	Ratio H_2_O : Zn : Al
Al-rich	0.20	0.15	0.15	4 : 3 : 3
Zn-deficient	0.20	0.125	0.125	4 : 2.5 : 2.5
Optimised	0.20	0.15	0.125	4 : 3 : 2.5
H_2_O-deficient	0.10	0.15	0.125	2 : 3 : 2.5

**Fig. 2:**
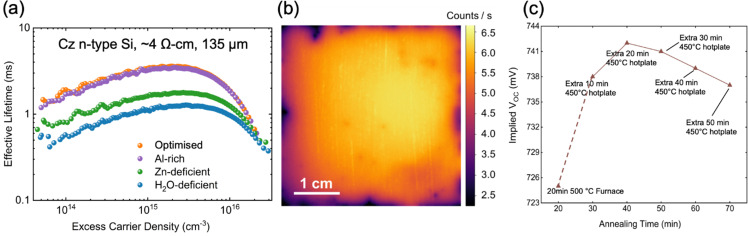
(a) Effective lifetime *vs.* excess carrier density of samples from the four different pressure profiles; (b) PL image of the champion sample from the optimised recipe; (c) iV_OC_ values *vs.* annealing time for the champion sample undergoing post-deposition annealing at 450 °C.

Fig. 2a shows the effective lifetime as a function of excess minority carrier density for the champion sample for each recipe. Each sample received a 20 min anneal at 500 °C, in a box furnace in ambient atmosphere, followed by a 10 min anneal on a hotplate at 450 °C. Table S1 (ESI†) displays the average and standard deviation iV_OC_ for 5 samples fabricated and measured per condition. Table S2 (ESI†) shows the extraction of *J*_0_ and iV_OC_ values (calculated using the models described in the ESI†) of the effective lifetime data on the champion samples from each profile.

The optimised passivation performance was achieved with a precursor pressure ratio H_2_O : Zn : Al of 4 : 3 : 2.5, represented by the orange curve in Fig. 1b. The corresponding lifetime for the optimised ratio at 10^15^ cm^−3^ excess carrier density (ECD) is 3.38 ms. The ALD recipe used to synthesise this sample is subsequently referred to as ‘optimised’ since recipes were optimised for carrier lifetime. Fig. 2b displays a photoluminescence image for the champion sample for the optimised recipe, indicating acceptable uniformity of the achieved passivation. We note that a common reason for deficient passivation in a given sample could result from a lack of spatial uniformity across the sample area, arising from contamination in the ALD chamber. Fig. 2c illustrates the effect of post annealing at 450 °C, on a hotplate, after the initial 500 °C anneal for 20 min in a box furnace. At the hotplate annealing time of 20 minutes (following the furnace step), the maximum iV_OC_ of above 741 mV was observed, while the iV_OC_ after 10 minutes was marginally lower, at 738 mV, with this step providing the most significant increase in iV_OC_. 10 minutes of hotplate annealing was selected for the standard recipe as it allowed the rapid synthesis of samples for improved statistics, with iV_OC_ values only marginally lower than the maximum achieved. We can also observe from Fig. 2c that longer hotplate annealing degrades the iV_OC_ above the annealing time of 20 minutes. Possible explanations include the hydrogen effusion from samples or the growth of ZnO crystallites as reported for ALD TiO_*X*_.^39^ The lifetime curves were modelled to extract the surface recombination current density (*J*_0s_) for each specimen. A *J*_0s_ of 0.87 fA cm^−2^ on each side was achieved for the optimised recipe. The effective lifetime and *J*_0s_ as a function of ECD is plotted in Fig. S1a and b (ESI†), while the extracted iV_OC_ is shown in Fig. S1c (ESI†). A corresponding iV_OC_ is among the highest recorded for AZO passivation schemes, while the *J*_0_ is the lowest recorded.^40–42^ We note that our wafers are industrially compatible 135 mm thick n-type silicon. The passivation mechanism of the optimised sample was investigated further using surface photo-voltage measurements. Fig. S2 (ESI†) shows a modest negative charge concentration in the region of 1 × 10^11^–1 × 10^12^ q cm^−2^, fitting with the band bending extracted from the XPS discussed in the next section. To achieve a lifetime value of >3 ms, a defect density at the mid bandgap of *D*_it,mg_ < 1 × 10^11^ cm^−2^ is expected.^43,44^

The Al-rich recipe achieved a lifetime similar to the optimised recipe, with an identical iV_OC_ of 740 mV, a lower lifetime at 10^15^ cm^−3^ ECD of 3.25 ms, and a slightly higher *J*_0_ of 0.88 fA cm^−2^. However, changing the recipe to be either Zn-deficient or H_2_O-deficient has a significant impact on the lifetime. The corresponding lifetime for these cases at 10^15^ cm^−3^ ECD was 1.62 ms and 1.12 ms, respectively. The iV_OC_ was 733 mV and 727 mV for Zn-deficient or H_2_O-deficient recipes, respectively. Varying the precursor flow in the recipe impacts the amount of Al dopant incorporated into the film, which in turn may influence its structural and compositional properties. The differences in dopant concentration can affect the film's crystallinity, defect density, band bending, and overall uniformity, which may affect the passivation performance. Therefore, it is essential to examine how these variations alter the microstructure and elemental distribution, providing insight into the mechanisms that drive the observed differences in lifetime, *J*_0_, and iV_OC_ across the different recipes.

## Structure and chemical composition

**Fig. 3:**
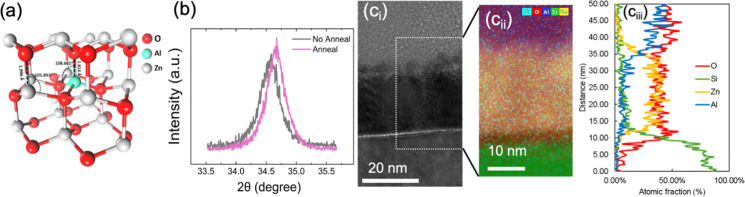
(a) Structural model of AZO. (b) XRD spectra of the AZO film in the range of 33.5–35.5° to give insight into the observed peak shift between not annealed and annealed sample; (c_i_) high-resolution high-angle annular dark field (HAADF) TEM image of the crystal structure and defects in AZO; (c_ii_) the enlarged image is the obtained EDS micrograph for a local region of interest. (c_iii_) The averaged EDS has been plotted for elemental analysis of AZO.

The structural and compositional characteristics of the AZO layers have been examined through a combination of X-ray diffraction (XRD), transmission electron microscopy (TEM), and energy-dispersive X-ray spectroscopy (EDS). Fig. 3a shows the hexagonal close-packed (hcp) structural model of AZO calculated from density functional theory (DFT), which aligns with the crystallographic results observed in the XRD spectra shown in Fig. 3b. The diffraction peak at 34.6° is characteristic of the (002) plane of ZnO for the well-known hexagonal closed-packed (hcp) structure. A slight shift in the diffraction peak is observed after annealing at 450 °C, towards a higher angle. This indicates a reduction in the lattice parameter, implying densification of the film, but otherwise the phase remains consistent before and after the annealing. This densification is likely due to the relaxation of internal stresses and possible redistribution of Al atoms within the ZnO matrix during annealing. Densification is also possible *via* the effusion of H_2_, H_2_O, and carbon species during post-annealing. Theeuwes *et al.* showed that such effusion occurs above about 200 °C in the case of ALD Al_2_O_3_ deposited at 100 and 200 °C.^45^

Fig. 3c_i_ presents a high-resolution transmission electron microscope (HRTEM) micrograph of a cross-section in the optimised recipe, showing the detailed nanolayer arrangement across the SiO_*x*_/AZO/AlO_*x*_ stack. The nano-crystallites are visible in the AZO and are embedded in an amorphous AZO matrix, indicating the possible presence of phase boundary defects, which could act as sites of charge trapping or scattering, affecting the electronic properties. The elemental distribution zoom-in map was obtained *via* EDS (Fig. 3c_ii_), revealing the spatial concentration of key elements: O (red), Si (green), Zn (yellow), and Al (blue). There is a consistent distribution of Zn, Al, and O within the AZO matrix, with sharp transitions at the c-Si interface, confirming high film uniformity. This film control is crucial for ensuring effective passivation and confirms successful Al incorporation *via* ALD. Atomic fraction profiles were obtained from the averaged EDS micrograph and are shown in Fig. 3c_iii_. The Al concentrations gradually taper off near the surface, indicating the well-distributed doping process in the AZO film.

The XRD and HRTEM results indicate high structural and compositional integrity for the optimised nanolayer AZO films. It is clear that the optimised ALD process (4 : 3 : 2.5 ratio of H_2_O : Zn : Al) leads to excellent film conformality, a continuous interfacial SiO_*x*_, and controlled Al doping. The XRD data suggests that the slight densification after annealing improves the crystallinity without altering the overall phase. Additionally, the uniform Al distribution observed in the elemental analysis is expected to deliver effective doping, which increases the *n*/*p* charge carrier ratio at the Si surface *via* the field effect mechanism, improving passivation.

To further elucidate the role of Al incorporation in AZO films, X-ray photoelectron spectroscopy (XPS) analysis was conducted to examine the Al concentration across different precursor pressure profiles. XPS allows for a more detailed investigation into the chemical composition and distribution of Al within the films, thereby complementing the microstructural insights from HRTEM and XRD. AZO films prepared for XPS were deposited to a thickness of 50 nm and annealed under the same conditions as samples for lifetime measurements, without the AlO_*x*_ capping layer so that the isolated AZO layer could be measured. XPS surveys (see Fig. S3, ESI†) exhibited Zn, O and Al core levels, as well as carbon, suggesting some surface contamination. The sample surfaces were then sputtered with an Ar^+^ ion gun and a second survey (see ESI†) showed the carbon peak had disappeared while the other core levels increased in intensity, indicating that surface contamination was removed but the AZO layer was not sputtered through.

**Fig. 4:**
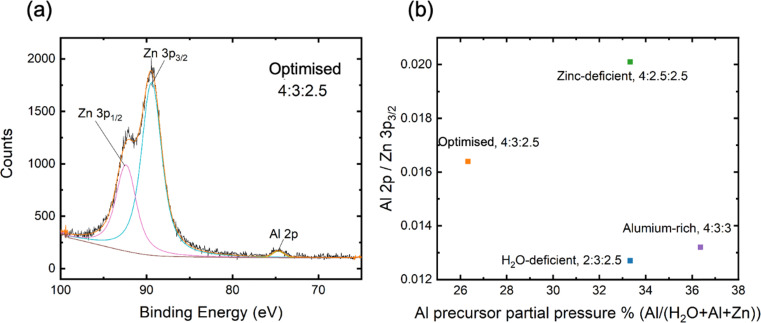
(a) Example of fitting of Zn 3p & Al 2p core level peaks in XPS spectra, for optimised case only; (b) Al 2p/Zn 3p_3/2_ ratio across annealed, sputter-cleaned samples with the four different pressure profiles, determined from the integrated intensity of Zn 3p_3/2_ and Al 2p core peaks, as measured with XPS.

The extracted Al 2p/Zn 3p_3/2_ ratios of core level intensities are shown in Fig. 4, demonstrating significant differences depending on the precursor purge pressures used. The core level peak fitting is shown for all samples in Fig. S4 (ESI†). These values could not be translated into absolute concentrations, as the relative sensitivity factors (RSF) for the measurement system were not known. EDS results would suggest these correspond to Al concentrations on the order of 5 at% while the difference in the ratio of the Zn and Al core levels is intended to provide a more robust fractional comparison between samples.

The XPS analysis demonstrates that the Al 2p/Zn 3p_3/2_ ratio does not linearly correlate with the partial pressure of the Al precursor, but rather is influenced more significantly by the Zn precursor purge pressure. Interestingly, the Zn-deficient sample, which exhibited the highest Al 2p/Zn 3p_3/2_ ratio, did not exhibit superior passivation (when compared to the effective lifetime curves in Fig. 2a), reinforcing the notion that optimised passivation is not merely a function of higher Al content but depends on a delicate balance between Zn and Al concentrations. Furthermore, the observation of a higher Al 2p/Zn 3p_3/2_ ratio after surface sputtering suggests that the bulk of the AZO film contains more Al than the air-exposed surface.

The combined structural, compositional, and chemical analyses provide a comprehensive picture of AZO chemistry. They suggest that effective passivation correlates with optimised Al doping, where both concentration and distribution within the ZnO matrix are relevant. The interaction between the Al and Zn precursors during deposition is crucial for tuning the material's properties, emphasizing the importance of precise ALD process control to optimise structural quality and passivation performance.

## Electrical, optical, and device properties of AZO nanolayer TCOs

**Fig. 5:**
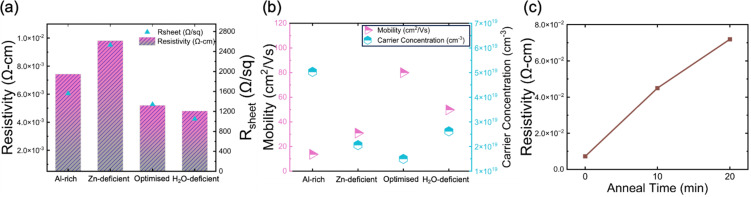
(a) Resistivity and sheet resistance and (b) mobility and carrier concentration of samples from the four different pressure profiles; (c) change in resistivity as a function of annealing time of the sample from the optimised pressure profile.

The electrical properties of AZO nanolayers were probed *via* van der Pauw (vdP) and Hall measurements. Fig. 5 presents the recorded resistivity, sheet resistance, mobility, and carrier concentration of AZO films with varying precursor pressure profiles, helping elucidate the influence of deposition conditions on electronic properties. For vdP-Hall measurements, 40-nm-thick AZO films were deposited *via* the same ALD processes on 290-nm-thick SiO_*x*_ on Si substrates without post-annealing process. The thickness of the samples for electrical measurements is twice the thickness for passivation measurements because the thinner 20 nm film did not allow surface electrical probing, causing unreliable and inconsistent results. The 290 nm SiO_*x*_ layer prevented the probe from penetrating through to the Si, which would affect the electrical results for AZO. The electrical measurements after the post-annealing process were also carried out for reference. Fig. 5a presents the resistivity (left axis, bars) and sheet resistance (right axis, triangles) for the four different pressure profiles. The optimised sample shows a resistivity of approximately 5 × 10^−3^ Ω-cm and sheet resistance of around 1300 Ω sq^−1^, indicating sufficient electronic transport for transparent conductor applications.^46^ The H_2_O-deficient samples have similar resistivity (4.8 × 10^−3^ Ω-cm) and sheet resistance (1100 Ω sq^−1^) as the optimised sample, suggesting that these moderate variations in precursor water content have a limited effect on electrical performance. The Al-rich sample shows higher resistivity and sheet resistance. This could arise from increased scattering or defects introduced by excess Al, leading to a poorer overall conductivity. Notably, the Zn-deficient sample exhibits the highest resistivity of 9.8 × 10^−3^ Ω-cm, with sheet resistance >2500 Ω sq^−1^. This significant increase in resistivity and sheet resistance highlights the critical role of controlling the Zn precursor dose in maintaining good electrical properties, which is in line with the XPS findings revealing that the Al concentration is not directly proportional to the partial pressure of the Al precursor, but more significantly affected by the purge pressure of the Zn precursor.

Hall effect measurements of AZO films with varying precursor pressure profiles were carried out in the same configuration as the vdP test. Fig. 5b shows the electron mobility (left axis, pink triangles) and carrier concentration (right axis, cyan hexagons). The optimised sample exhibits the highest mobility of approximately 80 cm^2^ V^−1^ s^−1^ and the lowest carrier concentration of 1.5 × 10^19^ cm^−3^. The Al-rich sample shows the lowest mobility and the highest carrier concentration. The H_2_O-deficient sample, compared to the Zn-deficient sample, exhibits higher carrier concentration. This likely arises from the reduction in oxygen precursor, which can increase oxygen vacancies, generate a large number of unpaired electrons on the surface, enhance the number of active sites, and thus increase the free carrier density.^47^

Fig. 5c illustrates the effect that annealing (450 °C, in air) has on the resistivity (measured by vdP) of the optimised sample. As the annealing time increases from 0 minutes (as-deposited) to 20 minutes, the resistivity steadily increases from 7 mΩ-cm to 72 mΩ-cm. This trend suggests that extended annealing might decrease free carrier concentration, which may be due to a reduction in oxygen vacancies.^48–50^ In our work, the AlO_*x*_ capping layer was not present to allow resistivity measurements. However, Macco *et al.* have found that, when capped by the AlO_*x*_, the resistivity does not degrade during post-deposition annealing.^32^

The Al-concentration (as estimated from XPS) does not directly correlate to the carrier density measured *via* the Hall effect, suggesting differences in dopant activation, and that the efficiency of Al incorporation and activation varies depending on the specific synthesis regime. This may indicate a relatively inefficient dopant incorporation process when using TMA rather than DMAI, as was reported by Macco *et al.*^37^ Although the impressive carrier lifetimes demonstrated in this work suggest that TMA may have advantages for surface passivation.

**Fig. 6:**
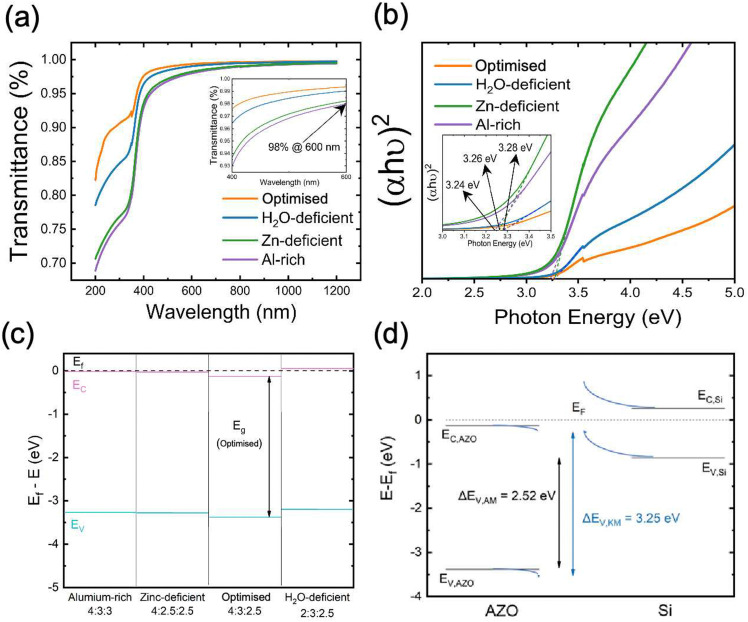
(a) Transmittance curves and (b) Tauc plots from optical band diagram of samples from the four different pressure profiles; (c) band diagram deduced from the combination of XPS and UV-vis; (d) valence band offset of optimised AZO sample and Si substrate, as determined from XPS measurements *via* the Anderson method (Δ*E*_V,AM_) from bulk valence band positions, and the Kraut method (Δ*E*_V,KM_) from valence band positions at the AZO/Si interface. Conduction band positions are inferred from bandgaps determined *via* UV-vis.

We also investigated the optical properties of AZO nanolayers by Ultraviolet–visible (UV-vis) spectrophotometry. Transmission curves are shown in Fig. 6a, covering the wavelength range from 200 nm to 1200 nm. For wavelengths >600 nm, the transmittance remains above 98% for all samples, indicating good transparency. Notably, the optimised sample (orange curve) demonstrates the highest transmittance, while the H_2_O-deficient sample (blue curve) shows reduced transmittance, particularly in the UV region (<400 nm). The Al-rich samples exhibit the lowest transmittance across the spectrum, which is in good correlation with high carrier concentration due to the many-body band gap renormalisation effect.^51,52^ In highly doped n-type semiconductors, such as the Al-rich AZO, an increase in free electron concentration fills the lower energy states of the conduction band. As a result, photons with lower energy (longer wavelengths) cannot excite electrons into the conduction band, effectively “shifting” the absorption edge to higher energies. Meanwhile, the high carrier concentration increases free carrier absorption, where free electrons interact with incident light and cause lower energy photons to be absorbed. This explains why the Al-rich sample, with its elevated electron density, shows the lowest optical transparency across the measured spectrum.

In Fig. 6b, Tauc plots are used to determine the direct optical bandgap of the AZO samples. The bandgap values are extracted by fitting the linear region of the plot (*αhν*)^2^*versus* photon energy and extrapolating to the intercept at (*αhν*)^2^ = 0. All the samples appear to have bandgaps clustered around 3.25 eV, with only slight variations. This suggests that while the precursor ratio affects the material properties, the differences in bandgap among these four samples are small. However, it can be observed from Fig. 6b that the Al-rich sample has the lowest measured optical bandgap of <3.25 eV.

Along with the valence band positions determined from XPS (see Fig. S5, ESI†) the band structures of each sample type (relative to the Fermi energy) are shown in Fig. 6c. The valence band positions are based on a linear extrapolation of the valence band edge from XPS, and the conduction band position is the inferred position from the optical bandgap values. Since these materials are expected to be degenerately-doped, we would expect that *E*_g_ = *E*_f_ − *E*_V_. For all cases but the optimised case, the bandgap inferred from XPS and the optical methods are aligned, implying degenerate doping, and in the case of the optimised sample, *E*_g,optical_ < *E*_f_ − *E*_V_, also consistent with degenerate doping, but possibly demonstrating the difference in measurement outcomes between absorption and XPS in the case of a partially-filled conduction band. The larger binding energy of the valence band compared to other samples suggests that the optimised sample is more highly doped. From this, we infer a larger offset between the valence bands of AZO and Si in the case of the optimised ALD recipe, which could produce a greater field-effect and so contribute to greater passivation. However, this result is inconsistent with the Hall effect results in Fig. 5b, where the carrier density was lowest in the optimised case and highest in the Al-rich case, and so does not provide a single answer for the improved passivation properties in the optimised case. Reasons for the inconsistency between valence band positions from XPS and carrier densities from the Hall effect could relate to a difference between annealed and non-annealed samples, a native space-charge region at the AZO top surface or Fermi-level shifts as a result of sputter damage-induced surface defects.

Estimates of the valence band offset between the AZO and the Si substrate in the case of the optimised lifetime sample are shown in Fig. 6d. Offsets were estimated from the XPS-extracted valence band maximum of the AZO, and the calculated valence band position for the Si wafer based on its known resistivity (4 Ω-cm) and dopant type (phosphorous). This yielded a valence band maximum 0.86 eV below the Fermi level. A direct measurement of the bulk Si valence band position with XPS was not considered as reliable as the calculation based on doping, since defects on the c-Si surface can significantly shift or pin the Fermi level^53^ and Fermi level pinning as a result of sputter damage has been reported in other photoemission studies.^54^ Taken together, these results implied a type II (staggered gap) alignment between the materials, with a valence band offset of 2.52 eV. Meanwhile, the offset as determined by the Kraut method^55^ (see ESI,† for calculation), with core level shifts at the interface used to determine band bending, implied a valence band offset of 3.25 eV, equal to the AZO bandgap measured by absorption, and so ambiguously suggesting either a type II or type III (broken gap) alignment. Time constraints prevented the measurement of all samples this way, meaning comparisons of the offset could not be made between samples Fig. 6.^53^ Regardless, a large valence band offset was observed between the AZO and Si, with direct interface measurements (Kraut method) indicating a significantly larger offset than implied by the bulk valence band maximum positions. This large offset may have a positive impact on field-effect passivation but could be detrimental to contact resistance. Comparisons of the offset could not be made across all sample types, but will be a priority for future studies.

The combined electrical and optical analyses reveal certain correlations between the carrier concentration, mobility, resistivity, and transmittance of AZO films and the precursor ratios used during their deposition. The Al-rich sample, with the highest carrier concentration, suffers from reduced mobility and lower optical transmittance, which may result from increased free carrier absorption and the many-body band gap renormalization effect. In contrast, the optimised sample balances carrier concentration and mobility, resulting in low resistivity and high transmittance, making it ideal for both transparent conductive and passivation applications. The Zn-deficient and H_2_O-deficient samples show higher carrier concentration, lower mobility, and lower optical performance than the optimised one, emphasising the importance of proper DEZ and H_2_O levels in optimising film properties. This relationship underscores the need to precisely control ALD conditions to tune AZO films for specific optoelectronic applications.

In addition to the precise control of AZO film fabrication conditions, the trade-off between conductivity and transparency regarding TCO thickness is a critical aspect of solar cells and other optoelectronic devices. Therefore, we investigated the AZO film resistivity under different thicknesses and included ESI,† in Fig. S6. Note that all the AZO films were deposited using the optimised recipe. It is clear that increasing thickness leads to increased conductivity.

**Fig. 7:**
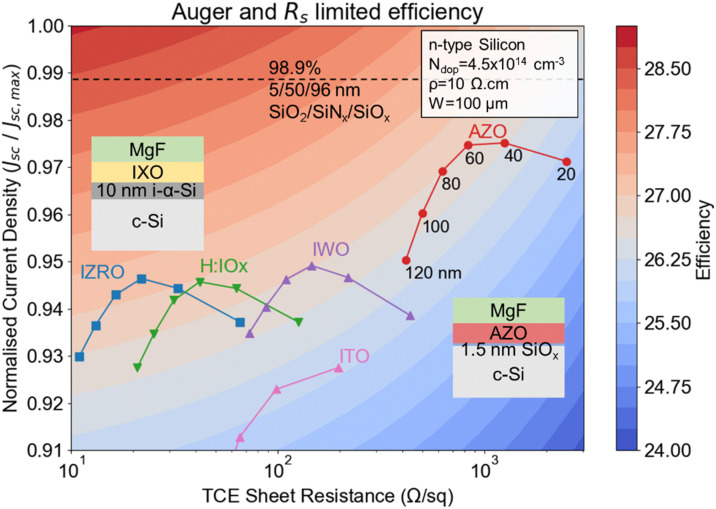
A plot of the maximum achievable efficiency of a silicon heterojunction cell, varying the sheet resistance and integrated transmittance of various TCE material across the AM 1.5G solar spectrum. The TCE materials include AZO, H:IOx, IWO, and IZRO.

From ellipsometry, we fitted the *n*, *k*, and thickness values of all the samples from different pressure profiles, as listed in Table S3 (ESI†). Fig. S7 (ESI†) illustrates the fitting curve for the optimised recipe. As shown in Table S3 (ESI†), ∼20 nm (Zn-deficient, optimised) and ∼23 nm AZO (Al-rich, H_2_O-deficient) samples were analysed in ellipsometry, leading to a variation of a magnitude of 10^−2^ in the *k* value at 600 nm wavelength. However, the total transmittance metric does not comprehensively describe device performance as it disregards the interference effects. Therefore, we simulate and find optimised thicknesses for AZO layers that account for all-optical phenomena. Using *n* and *k* values from Table S2 (ESI†), we simulated the expected optimised *J*_sc_ with AM 1.5G illumination and 100% EQE, with all simulation details provided in the ESI.†Fig. 7 presents a contour plot of the maximum achievable efficiency for a silicon heterojunction cell, incorporating different TCEs, assuming only optical, Auger and series resistance losses are present. Due to varying optical coupling at different TCE thicknesses, a MgF anti-reflection layer is incorporated to provide a just comparison between TCEs.

The *x*-axis shows the TCO sheet resistance (Ω sq^−1^), while the *y*-axis displays the normalised short-circuit current density (*J*_sc_/*J*_sc,max_). A variety of TCEs, including AZO, hydrogen-doped indium oxide (H:IOx),^56^ IWO,^57^ zirconium oxide-doped indium oxide (IZRO),^58^ ITO^59^ are evaluated, with varying thicknesses ranging from 20 to 120 nm. AZO films exhibit the best balance of sheet resistance and device current density at thicknesses near 60–80 nm, promising an efficiency potential of >26.5%. Modelled devices using IZRO, H:IOx, and IWO films demonstrate the strongest performance at thicknesses ∼60 nm as well, showing a good balance between device current density and resistance. Additionally, the model indicates that further reducing sheet resistance below 100 Ω sq^−1^ yields diminishing returns in terms of efficiency, where the films which maximise photon management, and thus current density, enable more substantial gains. This suggests that optimising both the TCE material and thickness is critical for maximizing solar cell performance, with AZO showing potential to achieve performance close to that of high mobility indium-based TCOs such as IZRO, and H:IOx, while matching that of IWO.

## Conclusions

We demonstrate that TMA can realise optimised surface passivation performance in Al-doped ZnO TCOs applied to n-type c-Si, while retaining excellent TCO properties. By optimising the ALD precursor dosing, we find that a pressure ratio of H_2_O : Al : Zn = 4 : 3 : 2.5 leads to exceptional surface passivation, with a *J*_0_ of 0.87 fA cm^−2^ and iV_OC_ of 740 mV. These are among the most efficient surface passivation metrics reported for a metal oxide that functions as a transparent, conductive and passivating material. Nanostructural characterisation revealed highly conformal and atomically controlled interfaces, with the presence of AZO nano crystallites bearing an hcp structure. The band diagram deduced from XPS and UV-vis measurements provides insight into the role that field-effect passivation plays for achieving high passivation from the AZO/AlO_*x*_ stack on Si. Field effect passivation, originating from TCO doping, electron affinity, and interface-charged states, provides a robust link between ALD precursor pressures and the resulting surface passivation. Overall, this work provides new insights that can help unlock and advance the use of earth-abundant TCO materials for TW production of efficient solar cells.

## Materials and methods

### Sample preparation

For lifetime measurement, we used phosphorus-doped n-type Cz crystalline silicon (c-Si) wafers with a resistivity of ∼4 Ω-cm and a thickness of 135 μm. These were laser-cut into 4 cm × 4 cm samples using a 1064 nm IR laser (Linxuan LX-A1-20W). After cutting, the samples were etched with tetramethylammonium hydroxide (TMAH) to remove saw damage, then etched in HF to remove surface oxide. Samples were cleaned using the standard Radio Corporation of America (RCA1 and RCA2) procedure,^60^ followed by a second HF etch and additional RCA2 step, which resulted in the formation of an approximately 1.2 nm thick SiO_*x*_ layer on the surface. The cleaned wafers were then placed into a Veeco Savannah thermal ALD chamber to deposit the AZO layer followed by the AlO_*x*_ capping layer. As precursors, we used widely available TMA, DEZ, and deionized (DI) water. The deposition process was carried out at 150 °C and a baseline pressure of 0.2 torr. The AZO layer, approximately 20 nm thick, was formed with a ZnO to AlO_*x*_ cycle ratio fixed at 20 : 1. The AlO_*x*_ capping layer was deposited to a thickness of approximately 25 nm. Deposition was performed on both sides of the wafer simultaneously. Following the deposition, the samples underwent post-treatment procedures: annealing at 500 °C for 20 minutes in air within a furnace, followed by a slow cool down for 10 minutes, followed by removal from the furnace. For passivation property tests, samples were annealed one more time at 450 °C for 10 minutes in air on a hotplate.

### Characterisation techniques

In this study, the microstructural characterization of the samples was conducted using an FEI Talos F200X TEM. The equipment operated at an accelerating voltage of 200 kV, equipped with a field emission gun (FEG) to provide a high-brightness electron beam. Imaging modes included both TEM and STEM modes. In TEM mode, the microscope's lattice resolution is better than 0.2 nm, allowing high-resolution imaging of the crystal structure and defects in the material. In STEM mode, HAADF imaging was performed, enabling Z-contrast imaging based on atomic number, with a resolution of approximately 1 nm. Data acquisition and processing were done using the associated Velox software to ensure image quality and the accuracy of subsequent analyses. Additionally, EDS was employed for elemental analysis to investigate the composition and distribution of elements in the samples. The thin sections of the samples were prepared using focused ion beam (FIB) technology to ensure the sample thickness met the transmission requirements. These techniques were performed in the Thermo Fisher Scientific Inc. Talos F200X scanning/transmission electron microscope (S/TEM) carried out by Jiangsu Institute of Advanced Semiconductors Ltd.

The thickness of the thin films was measured using ellipsometry (JA Woollam). The modelling parameters used for AZO were refractive index (*n*) ranging from 1.6 to 2.1, and extinction coefficient (*k*) ranging from 0 to 0.4 from 210 to 1690 nm wavelength. XRD (*θ*–2*θ*) measurements were carried out using a Rigaku miniflex with a Cu K-α_1_ source (1.541 nm wavelength), over a range of *θ* = 10–90°, at 0.05° per second.

XPS was employed to analyse the elemental ratio of Al and Zn in the films based on the integrated intensity of the Zn 3p and Al 2p core level peaks. Monochromated XPS measurements of the core levels were carried out with an Al Kα X-ray source (1486.6 eV), using a hemispherical analyser with the pass energy set to 26 eV. The Al 2p core level was observed to be weak in all samples, consistent with the expected Al concentrations of a few at%. The Al 2p/Zn 3p_3/2_ ratio was estimated from spectra taken after sputter cleaning, by fitting the core level peaks using CasaXPS to extract the integrated intensity, with the fits shown in Fig. S4 (ESI†). Translating these values to an Al concentration requires knowledge of the RSF which is specific to the XPS instrument used. Because RSF values were not available for the spectrometer used for in this work, the absolute values of concentration could not be known in this case. However, the trends in Al 2p/Zn 3p_3/2_ ratios between the samples should nevertheless be representative. The ESI,† explores the impacts of sputtering on the estimated Al 2p/Zn 3p_3/2_ ratios (Fig. S8, ESI†), but due to the possibility of surface contamination, sputter cleaning of samples was considered necessary for accurate results.

The valence band offsets could be estimated from the Kraut method by directly measuring the band bending at the AZO/Si interface. The interface was characterised by *in situ* sputtering through the AZO layer at 4 kV for 15 min using an Ar^+^ ion gun, such that a thin (∼1 nm) layer of AZO remained on the Si surface, thin enough that photoelectrons from the Si 2p core level escape through the AZO layer to be measured (Fig. S9, ESI†). In this interface region, the Si and Zn core levels are expected to shift compared to their bulk values by an energy equal to the shift in Si and AZO valence bands at the interface^55^ (the exact calculation is described in the ESI,† eqn (S1)). Therefore, we measured the position of the Zn 3p and Si 3p core levels at this interface region. The AZO valence band position at the interface was determined from the bulk AZO valence band, shifted by the difference between the bulk and interface Zn 3p core levels. Meanwhile, the Si interface valence band position was determined by adding a fixed value of 98.95 eV to the binding energy of the Si 2p core level, as this is well-defined for Si.^61^ The bulk valence band for the Si was then determined from its known doping level using tools available on PVLighthouse.^62^ The difference between the interface and bulk valence band positions corresponded to the interface band bending, and provided an estimate of the valence band offset between AZO and Si that took account of this phenomenon. This was only performed for the optimised lifetime sample, due to constraints on equipment access which prevented measurement of all sample types.

To characterise charge concentration in the AZO layer the surface photovoltage (SPV) was measured using a KP Technology Scanning Kelvin Probe (SKP5050) and a 10 mW cm^−2^ halogen lamp. A 1 × 1 cm map containing 36 points was measured using a 2 mm gold probe. The analysis of the charge was carried out using software available at https://github.com/OxfordInterfacesLab/. To complement the SPV measurements, the band bending at the Si surface was extracted using the Si 2p core level spectra obtained from XPS.

PCD lifetime measurements were conducted at room temperature using a Sinton WCT-120 lifetime tester, which were operated in an ambient air environment. In addition, PL imaging was utilised to evaluate the surface passivation uniformity of the AZO/AlO_*x*_ stacks. The sheet resistance of AZO was measured using the van der Pauw method with gold metal probes in direct contact with the AZO surface, as close to the edge as possible. Under a perpendicular magnetic field of 0.3 T, carrier concentration and mobility were determined by measuring the Hall effect technique. For both van der Pauw and Hall effect measurements, signal generation and data acquisition were conducted using a Keysight B2901A Source Measuring Unit.

A Cary 5000 UV-vis spectrophotometer was utilised to investigate the optical properties of the AZO, from which the optical band gap was estimated using a Tauc plot. Tauc plots of the film's optical absorption were constructed to extract optical bandgaps, which were then combined with XPS results to create a complete model of the electronic band alignment at AZO-Si interfaces. Transmission measurements were carried out on single-sided 40-nm-thick AZO samples deposited on fused quartz substrates (to enable measurements at UV wavelengths) over a wavelength range of 200 to 1200 nm (to capture the relevant spectral features).

## Data availability

The data supporting this article have been included as part of the ESI.† All data created during this research and published in this article is openly available from the Oxford University Research Archive and can be downloaded free of charge from https://ora.ox.ac.uk.

## Author contributions

The manuscript was written through contributions of all authors. All authors have approved the final version of the manuscript.

## Conflicts of interest

There are no conflicts to declare.

## Supplementary Material

YA-004-D4YA00552J-s001

## References

[cit1] Burrington J. D. (2024). Renewable Energy technical potential performance for zero carbon emissions. ACS Omega.

[cit2] Haegel Nancy M. (2023). *et al.*, Photovoltaics at multi-terawatt scale: Waiting is not an option. Science.

[cit3] Ember, Global Electricity Review 2023. https://ember-energy.org/latest-insights/global-electricity-review-2023/, (accessed February 5, 2025)

[cit4] Benda V., Cerna L. (2020). PV cells and modules – State of the art, limits and trends. Heliyon.

[cit5] Chavan G. T. (2023). *et al.*, A Brief Review of Transparent Conducting Oxides (TCO): The Influence of Different Deposition Techniques on the Efficiency of Solar Cells. Nanomaterials.

[cit6] Ru X. (2024). *et al.*, Silicon heterojunction solar cells achieving 26.6% efficiency on commercial-size p-type silicon wafer. Joule.

[cit7] Lin H. (2023). *et al.*, Silicon heterojunction solar cells with up to 26.81% efficiency achieved by electrically optimized nanocrystalline-silicon hole contact layers. Nat. Energy.

[cit8] Yu C. (2023). *et al.*, Industrial-scale deposition of nanocrystalline silicon oxide for 26.4%-efficient silicon heterojunction solar cells with copper electrodes. Nat. Energy.

[cit9] Yu C. (2023). *et al.*, Silicon solar cell with undoped tin oxide transparent electrode. Nat. Energy.

[cit10] Wright M. (2023). *et al.*, Design considerations for the bottom cell in perovskite/silicon tandems: a terawatt scalability perspective. Energy Environ. Sci..

[cit11] Zhang Y. (2021). *et al.*, Design considerations for multi-terawatt scale manufacturing of existing and future photovoltaic technologies: challenges and opportunities related to silver, indium and bismuth consumption. Energy Environ. Sci..

[cit12] Wagner L. (2024). *et al.*, The resource demands of multi-terawatt-scale perovskite tandem photovoltaics. Joule.

[cit13] Al-Arique H. Q. N. M. (2024). *et al.*, Study the characterization of ZnO and AZO films prepared by spray pyrolysis and the effect of annealing temperature. Opt. Mater..

[cit14] Wai H. S., Li C. (2022). Effect of Aluminum Doping Ratios on the Properties of Aluminum-Doped Zinc Oxide Films Deposited by Mist Chemical Vapor Deposition Method Applying for Photocatalysis. Nanomaterials.

[cit15] Gibson K., Johlin E., Yang D. (2024). Improved perovskite photostability via application of TiO2, ZnO and AZO thin films by pulsed laser deposition. Opt. Mater..

[cit16] Kek R. (2020). *et al.*, Effects of background gases and pressure in pulsed laser deposition of Al-doped ZnO. Thin Solid Films.

[cit17] Van Toan N. (2021). *et al.*, Aluminum doped zinc oxide deposited by atomic layer deposition and its applications to micro/nano devices. Sci. Rep..

[cit18] Zhao K. (2022). *et al.*, Investigation on Transparent, Conductive ZnO:Al Films Deposited by Atomic Layer Deposition Process. Nanomaterials.

[cit19] Alev O. (2024). *et al.*, Effect of Al doping on structural and optical properties of atomic layer deposited ZnO thin films. Surf. Interfaces.

[cit20] Singh R., Mukherjee S. K. (2023). RF sputtered AZO thin films: A potential TCO for various opto-electronic applications. Mater. Today.

[cit21] Challali F. (2020). *et al.*, Effect of RF sputtering power and vacuum annealing on the properties of AZO thin films prepared from ceramic target in confocal configuration. Mater. Sci. Semicond. Process..

[cit22] Khan M. I., Neha T. R., Billah M. M. (2022). UV-irradiated sol-gel spin coated AZO thin films: enhanced optoelectronic properties. Heliyon.

[cit23] Gupta B. (2021). *et al.*, Recent Advances in Materials Design Using Atomic Layer Deposition for Energy Applications. Adv. Funct. Mater..

[cit24] Zhang J. (2022). *et al.*, Advances in Atomic Layer Deposition. Nanomanuf. Metrol..

[cit25] Sinton R. A., Cuevas A. (1996). Contactless determination of current-voltage characteristics and minority-carrier lifetimes in semiconductors from quasi-steady-state photoconductance data. Appl. Phys. Lett..

[cit26] Green M. (2020). *et al.*, Solar cell efficiency tables (version 57). Prog. Photovoltaics Res. Appl..

[cit27] Battaglia C., Cuevas A., De Wolf S. (2016). High-efficiency crystalline silicon solar cells: status and perspectives. Energy Environ. Sci..

[cit28] Glunz S. W., Feldmann F. (2018). SiO_2_ surface passivation layers – a key technology for silicon solar cells. Solar Energy Mater. Solar Cells.

[cit29] Richter A. (2012). *et al.*, Improved quantitative description of Auger recombination in crystalline silicon. Phys. Rev. B:Condens. Matter Mater. Phys..

[cit30] Schmidt J., Peibst R., Brendel R. (2018). Surface passivation of crystalline silicon solar cells: Present and future. Solar Energy Mater. Solar Cells.

[cit31] Panigrahi J. (2017). *et al.*, Crystalline silicon surface passivation by thermal ALD deposited Al doped ZnO thin films. AIP Adv..

[cit32] Macco B. (2021). *et al.*, Atomic-layer-deposited Al-doped zinc oxide as a passivating conductive contacting layer for n + -doped surfaces in silicon solar cells. Solar Energy Mater. Solar Cells.

[cit33] Wu S.-H. (2017). *et al.*, A Design Based on a Charge-Transfer Bilayer as an Electron Transport Layer for Improving the Performance and Stability in Planar Perovskite Solar Cells. J. Phys. Chem. C.

[cit34] Chen W. (2022). *et al.*, N-type polysilicon passivating contact combined with hydrogen-containing TCO as the interconnected structure for perovskite/silicon tandem solar cells. Solar Energy Mater. Solar Cells.

[cit35] Ramírez-Esquivel O. Y. (2021). *et al.*, Atomic layer deposition supercycle approach applied to the Al-doping of nearly saturated ZnO surfaces. Ceram. Int..

[cit36] Niemelä J.-P. (2019). *et al.*, Rear-emitter silicon heterojunction solar cells with atomic layer deposited ZnO:Al serving as an alternative transparent conducting oxide to In2O3:Sn. Solar Energy Mater. Solar Cells.

[cit37] Wu Y. (2013). *et al.*, Enhanced Doping Efficiency of Al-Doped ZnO by Atomic Layer Deposition Using Dimethylaluminum Isopropoxide as an Alternative Aluminum Precursor. Chem. Mater..

[cit38] Gao Z., Banerjee P. (2019). Review Article: Atomic layer deposition of doped ZnO films. J. Vac. Sci. Technol., A.

[cit39] Nakagawa Y. (2020). *et al.*, Effect of forming gas annealing on hydrogen content and surface morphology of titanium oxide coated crystalline silicon heterocontacts. J. Vac. Sci. Technol., A.

[cit40] Cai H. (2024). *et al.*, Design and Optimization of SiOx/AZO Transparent Passivating Contacts for High-Efficiency Crystalline Silicon Solar Cells. Adv. Funct. Mater..

[cit41] Xie A. (2024). *et al.*, Bifacial silicon heterojunction solar cells using transparent-conductive-oxide- and dopant-free electron-selective contacts. Prog. Photovoltaics Res. Appl..

[cit42] Gao K. (2025). *et al.*, Efficient Silicon Solar Cells with Aluminum-Doped Zinc Oxide-Based Passivating Contact. Adv. Funct. Mater..

[cit43] Bonilla R. S. (2022). Modelling of Kelvin Probe Surface Voltage and Photovoltage in Dielectric-Semiconductor Interfaces. Mater. Res. Exp..

[cit44] Bonilla R. S., Wilshaw P. R. (2017). On the c-Si/SiO2 interface recombination parameters from photo-conductance decay measurements. J. Appl. Phys..

[cit45] Theeuwes R. J. (2023). *et al.*, Hydrogenation of p+ poly-Si by Al2O3 nanolayers prepared by atomic layer deposition. J. Appl. Phys..

[cit46] Aydin E. (2023). *et al.*, Enhanced optoelectronic coupling for perovskite/silicon tandem solar cells. Nature.

[cit47] Zhang C. (2020). *et al.*, Metal oxide semiconductors with highly concentrated oxygen vacancies for gas sensing materials: A review. Sens. Actuators, A.

[cit48] Luka G. (2011). *et al.*, The uniformity of Al distribution in aluminum-doped zinc oxide films grown by atomic layer deposition. Mater. Sci. Eng. B.

[cit49] Hsu C. H. (2020). *et al.*, Air Annealing Effect on Oxygen Vacancy Defects in Al-doped ZnO Films Grown by High-Speed Atmospheric Atomic Layer Deposition. Molecules.

[cit50] Zhou X. (2020). *et al.*, The Effects of Post Annealing Process on the Electrical Performance and Stability of Al-Zn-O Thin-Film Transistors. IEEE Electron Device Lett..

[cit51] Lu J. G. (2007). *et al.*, Carrier concentration dependence of band gap shift in n-type ZnO:Al films. J. Appl. Phys..

[cit52] Klein J. (2023). *et al.*, Limitations of the Tauc Plot Method. Adv. Funct. Mater..

[cit53] Himpsel F. J. (1988). *et al.*, Microscopic structure of the SiO2/Si interface. Phys. Rev. B: Condens Matter..

[cit54] Tsui B.-Y. (2017). *et al.*, Strong Fermi-level pinning induced by argon inductively coupled plasma treatment and post-metal deposition annealing on 4H-SiC. Solid-State Electron..

[cit55] Kraut E. A. (1980). *et al.*, Precise Determination of the Valence-Band Edge in X-Ray Photoemission Spectra: Application to Measurement of Semiconductor Interface Potentials. Phys. Rev. Lett..

[cit56] Macco B. (2014). *et al.*, High mobility In_2_O_3_:H transparent conductive oxides prepared by atomic layer deposition and solid phase crystallization. Phys. Status Solidi.

[cit57] Han C. (2021). *et al.*, Room-temperature sputtered tungsten-doped indium oxide for improved current in silicon heterojunction solar cells. Solar Energy Mater. Solar Cells.

[cit58] Aydin E. (2019). *et al.*, Zr-Doped Indium Oxide (IZRO) Transparent Electrodes for Perovskite-Based Tandem Solar Cells. Adv. Funct. Mater..

[cit59] Holman Z. C. (2013). *et al.*, Infrared light management in high-efficiency silicon heterojunction and rear-passivated solar cells. J. Appl. Phys..

[cit60] Kern W. (1990). The Evolution of Silicon Wafer Cleaning Technology. J. Electrochem. Soc..

[cit61] Man G. (2016). *et al.*, Electronically Passivated Hole-Blocking Titanium Dioxide/Silicon Heterojunction for Hybrid Silicon Photovoltaics. Adv. Mater. Interfaces.

[cit62] https://www.pvlighthouse.com.au, PV LightHouse. p. Cells calculators-Cells calculators, 2022

