# Supplementary material for: Impact of precursor dosing on the surface passivation of AZO/AlOx stacks formed using atomic layer deposition
Source: Energy Adv. 2025 Feb 3;4(4):553–64. doi: 10.1039/d4ya00552j (PMC11826515; doi:10.1039/d4ya00552j)
Supplement: YA-004-D4YA00552J-s001 [file YA-004-D4YA00552J-s001.pdf]

Supplementary Information (SI) for Energy Advances.
This journal is © The Royal Society of Chemistry 2025

Impact of precursor dosing on the surface passivation of AZO/Al_2O_3 stacks formed using atomic layer deposition

Supporting Information

Yan Wang[1], Theodore D.C Hobson[1], Jack E. N. Swallow[1], Shona McNab[2], John O'Sullivan[1], Anastasia H. Soeriyadi[1], Xinya Niu[1], Rebekah C. Fraser[3], Akash Dasgupta[3], Soumyajit Maitra[1], Pietro P. Altermatt[1], Robert S. Weatherup[1], Matthew Wright[1], Ruy S. Bonilla[1]*

1. Department of Materials, University of Oxford, Oxford, OX1 3PH, United Kingdom
2. University Of New South Wales, Sydney, NSW 2052, Australia
3. Department of Physics, University of Oxford, Oxford, OX1 3PU, United Kingdom

* Corresponding author: sebastian.bonilla@materials.ox.ac.uk

Table S1. IV_{OC} values for 5 samples per condition in group A,B,C, D with the average and standard deviation values calculated. (A, B, C, D represent Al-rich, Zn-deficient, optimised, and H2O-deficient, respectively)

iV_{OC} (mV)	Group A	Group B	Group C	Group D
**Sample 1**	731	717	738	717
**Sample 2**	732	726	740	706
**Sample 3**	740	728	735	723
**Sample 4**	740	731	739	726
**Sample 5**	740	732	740	719
**Average**	736.6	726.8	738.4	718.2
**Standard deviation. P**	4.18	5.34	1.85	6.85

Table S2. J0, iVoc, and lifetime (@ 1E15 ECD) values of all the champion samples in group A,B,C,D. (A, B, C, D represent Al-rich, Zn-deficient, optimised, and H2O-deficient, respectively)

	Group A	Group B	Group C	Group D
**J_0 (fA/cm^2)**	0.88	2.8	0.87	1.81
**iV_{OC} (mV)**	740	733	740	727
**Lifetime (ms) @ 1E15**	3.25	1.62	3.38	1.12

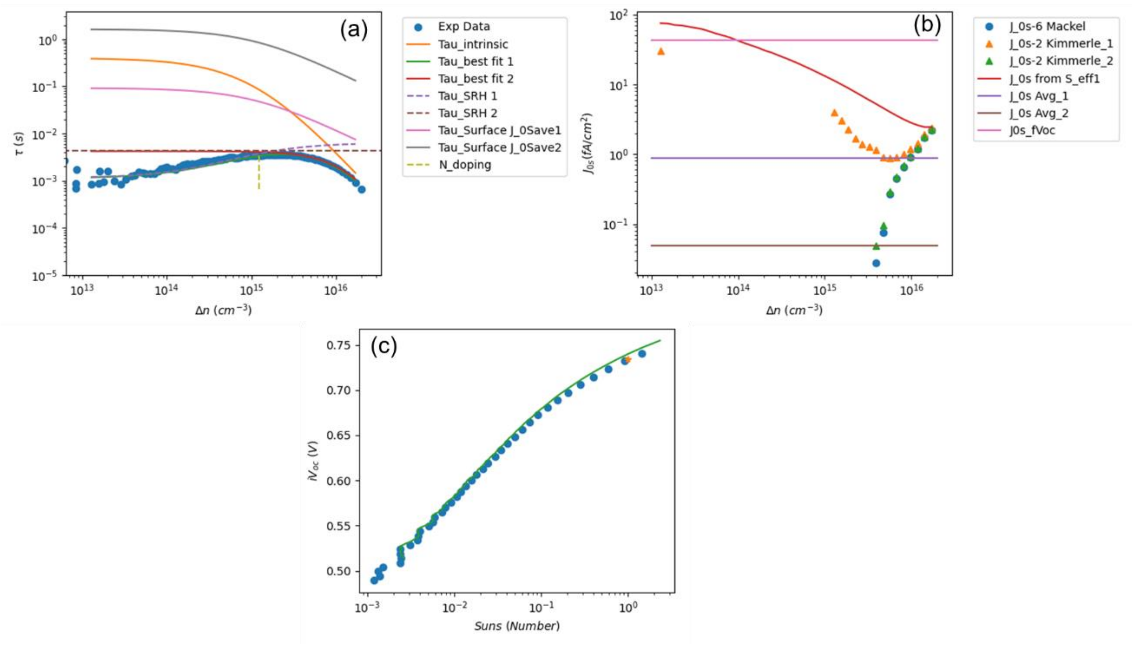

Figure S1. (a) Effective lifetime, (b) J0 and (c) iV_{OC} analysis plots for the highest Teff vs. Δn of samples in profile C (Optimised). Analysis is conducted following the algorithm and code reported in github.com/OxfordInterfacesLab/PySintonAnalysis

Table S3. n, k, and thickness of samples from the four different pressure profiles measured from ellipsometry. Data is acquired at 600 nm wavelength. (A, B, C, D represent Al-rich, Zn-deficient, optimised, and H2O-deficient, respectively)

Group	n	k	Thickness (nm)	Mean Squared Error (MSE)
A	1.79	0.011	23.85±0.25	32
B	1.74	0.003	19.38±0.25	24
C	1.76	0.020	19.85±0.27	28
D	1.77	0.036	22.76±0.58	55

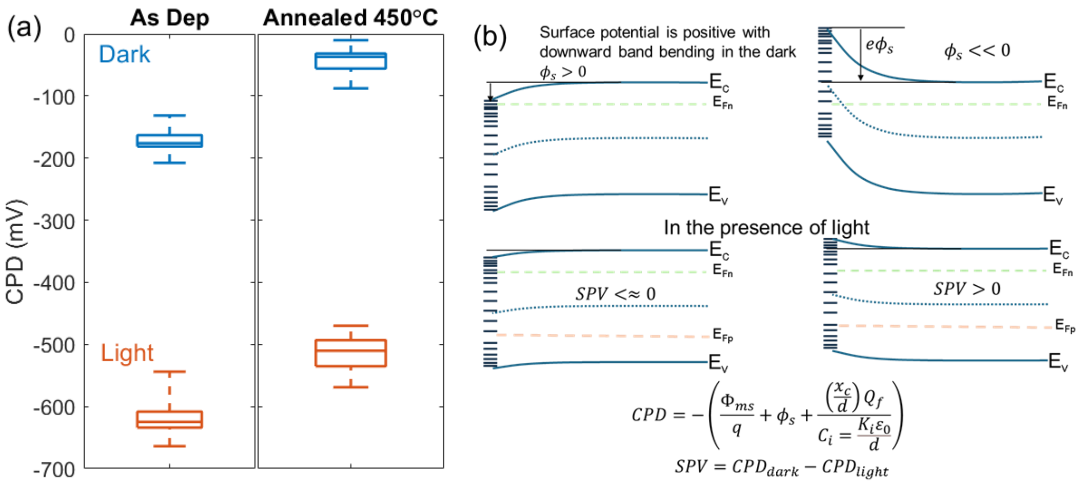

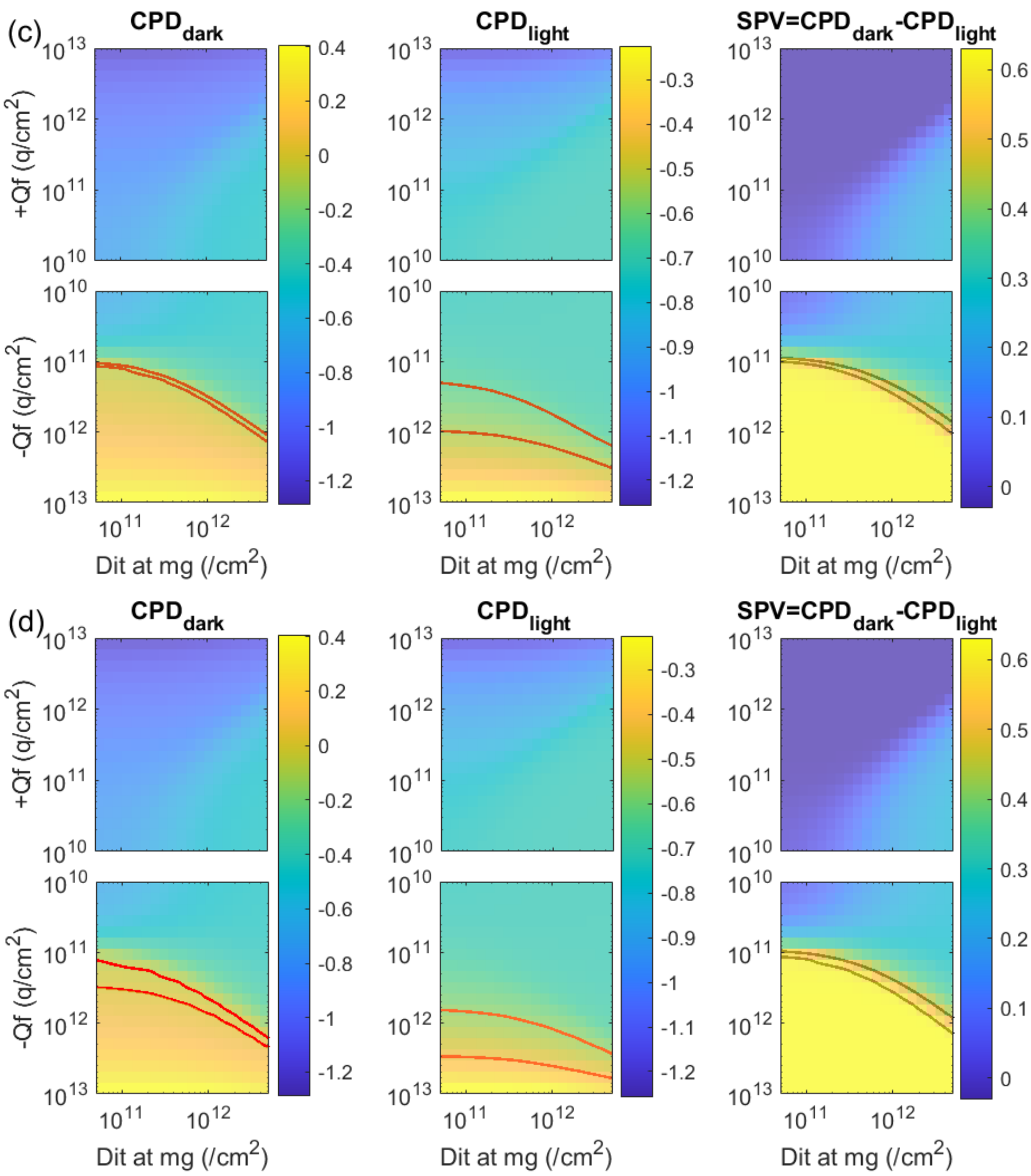

Figure S2. (a) Surface photovoltage (SPV) contact potential difference (CPD) measurements using Kelvin Probe on nSi/AZO as deposited and after a 450 °C hotplate anneal showing a negative value of SPV for both samples. (b) Schematic representation of CPD measured in the KP instrument, and the extracted SPV linking to the polarity of charge. Where, Φms is the work function difference between the gold probe and the silicon, q is the electron charge, ϕs is the surface potential, xc is the distance of the charge centroid from the interface, d is the AZO thickness, Qf is the fixed charge density, Ci is the capacitance generated from the interface defects and K-i is the AZO dielectric constant and ε0 is the permittivity of free space. c) and d) Analysis of SPV data to determine the approximate Qf and Dit for AZO as deposited and after anneal respectively. Both indicate a negative charge.

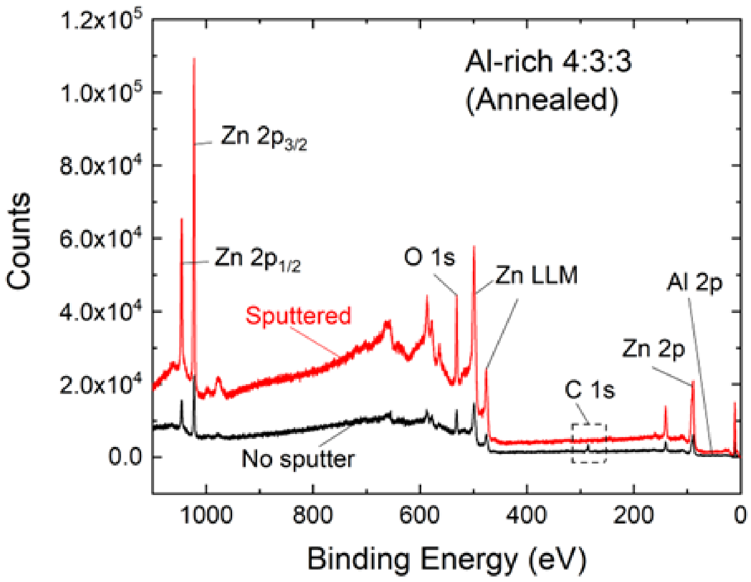

Figure S3. XPS survey spectra illustrating the effects of surface Ar sputtering at 4 kV for 5 mins, for annealed Al-rich sample. Spectra demonstrate increased signal intensity and removal of carbon 1s core level with sputtering, while AZO core levels remain unchanged, indicating effective removal of surface contaminants.

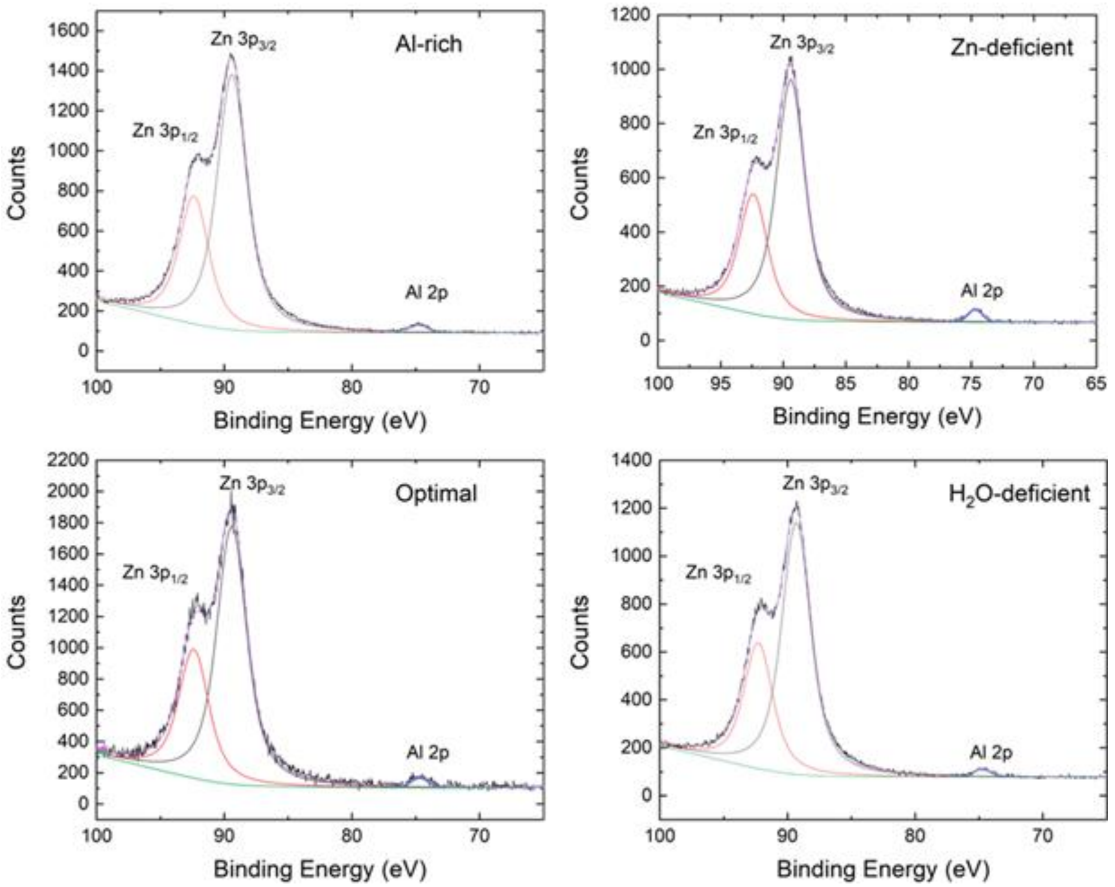

Figure S4. XPS spectrum range covering Zn $3p_{1/2}$ & $3p_{3/2}$ and Al 2p core levels for the 4 annealed samples, after surface sputtering, with fitted integrated intensity for Zn $3p_{3/2}$ and Al 2p in each case. Fitting was carried out in CasaXPS using Tougaard background and convolved Gaussian/Lorentzian lineshape. Zn $3p_{1/2}$ & $3p_{3/2}$ peaks were constrained to equal

FWHM with Zn $3p_{1/2}$ at 0.5x peak area of $3p_{3/2}$. Al 2p peaks could not be separately resolved so one peak was used to fit.

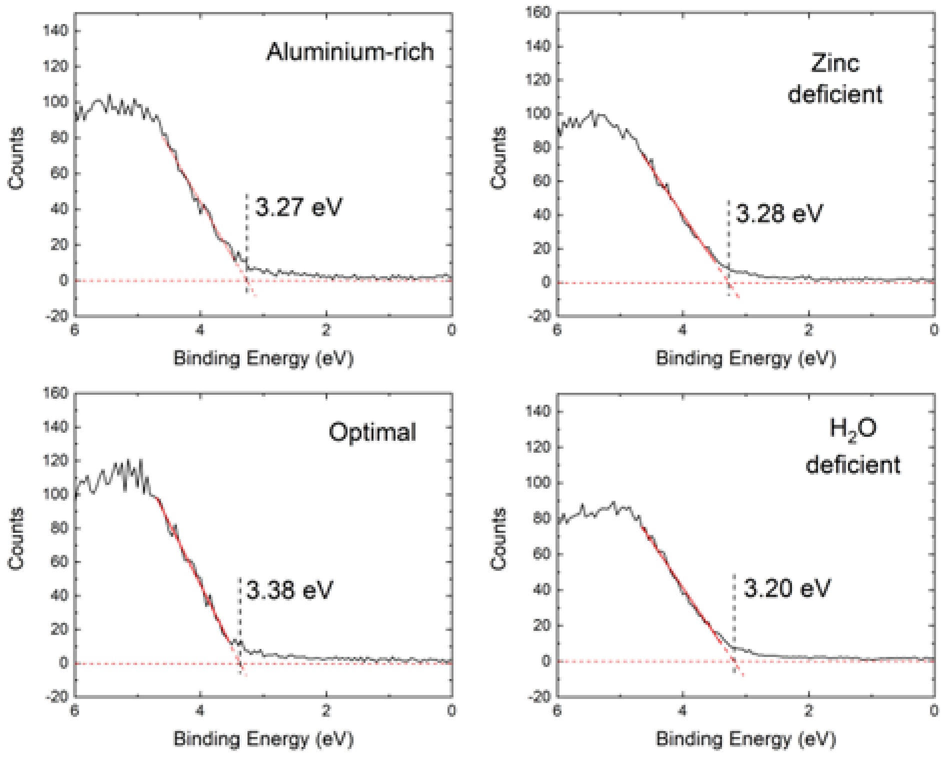

Figure S5. Valence band edges for samples after sputter-cleaning, with edge linearly fitted and valence band maximum determined from linear extrapolation to intercept at zero counts.

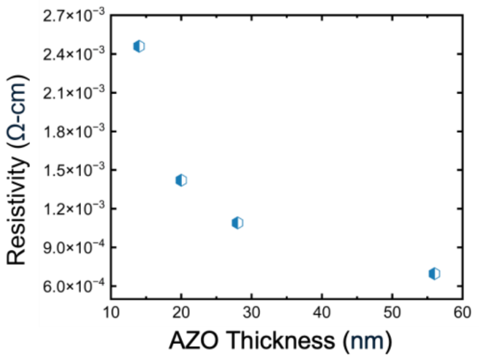

Figure S6. The resistivity of AZO thin films at different AZO thicknesses.

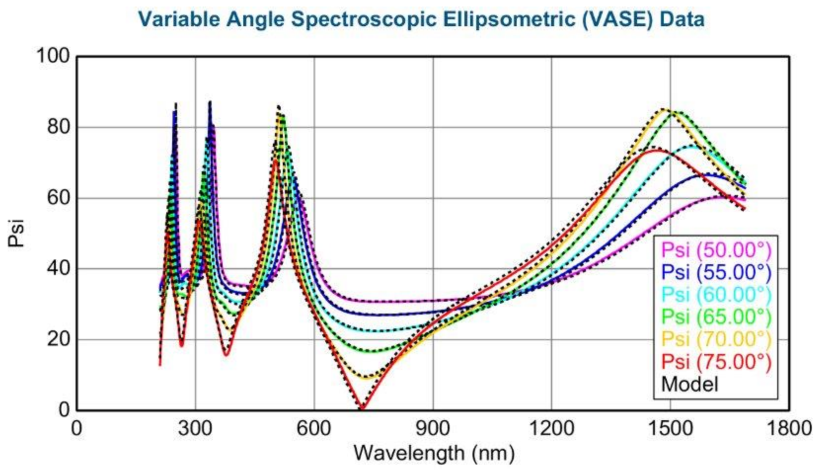

Figure S7. Fitting curve and the variable angle spectroscopic ellipsometric curves of the sample from pressure profile C.

The impact of sample surface sputtering upon Al-concentration is shown in **Figure S8**, where Al at.% increased for all samples after sputtering, suggesting a larger concentration in the bulk than at the air-exposed surface. The difference in Al-concentration between samples was consistent before and after sputtering, except in the case of the Al-rich sample, which exhibited larger Al at.% than the optimised sample before the sputter, but lower Al at.% afterwards.

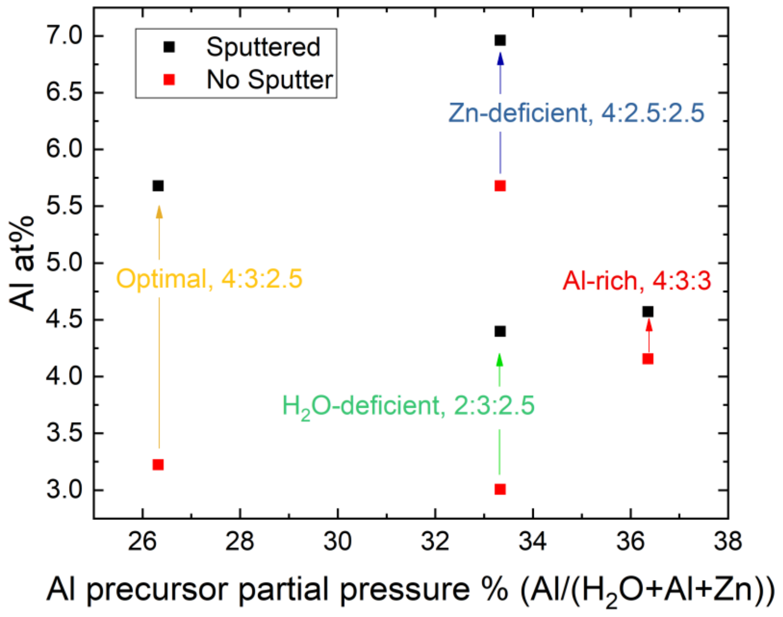

Figure S8. Comparison of estimated Al concentrations before and after sample surface sputtering, estimated from the ratio of Zn $3p_{3/2}$ and Al 2p peaks and RSF values from CasaXPS library.

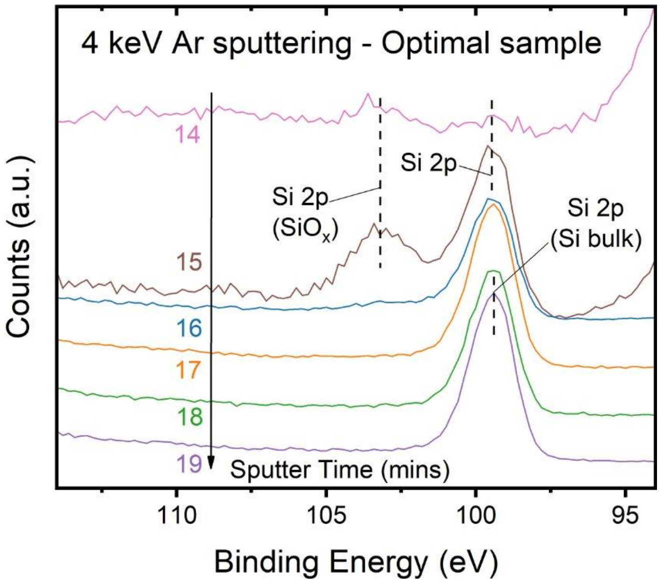

Figure S9. XPS spectra showing Si 2p core levels at the AZO/SiO_x/Si interface. AZO layer is thinned by sputtering with Ar gun (4keV) such that Si core levels can be detected and the shift in Si 2p and Zn 3p peaks relative to bulk positions allows band-bending to be estimated.

$$\Delta E_V = (E_{CL}^B - E_V^B) - \left(E_{CL}^A - E_V^A\right) + \Delta E_{CL}$$

Equation S1. ΔE_V is the measured valence band offset, E^B_{CL} is the position of a core level in the bulk for material B (e.g. AZO), E^B_V is the bulk valence band position for material B, E^A_{CL} is the position of a core level in the bulk for material B, E^A_V is the bulk valence band position for material A (e.g. Si) and ΔE_{CL} is the separation of the core levels as measured at the interface. When calculating the band bending, the 'bulk' value of the valence band maximum in Si was calculated based on the fixed binding energy difference between the Si 2p peak and the valence band of 98.95 eV, as reported by Man et al. [1]. When calculating the overall band bending, a bulk valence band maximum for the Si was calculated from the known resistivity of 4 Ω-cm and known dopant (phosphorous). The 'resistivity' and 'bandgap' calculator tools on PVLighthouse [2] were used to determine this value, placing the Fermi level 0.86 eV above the valence band maximum: https://www.pvlighthouse.com.au/resistivity, https://www.pvlighthouse.com.au/bandgap.

Modelling Losses from Resistance in TCEs

The resistive power loss due to the lateral current flow through the TCE at one side of the cell is:

$$dP = I^2 dR$$

Where *dR* is the resistance contributed by a *dx* section of the TCE as indicated in **Figure S9**, and given by:

$$dR_{TCE} = R_{sheet}^{TCE} \cdot \frac{dx}{b}$$

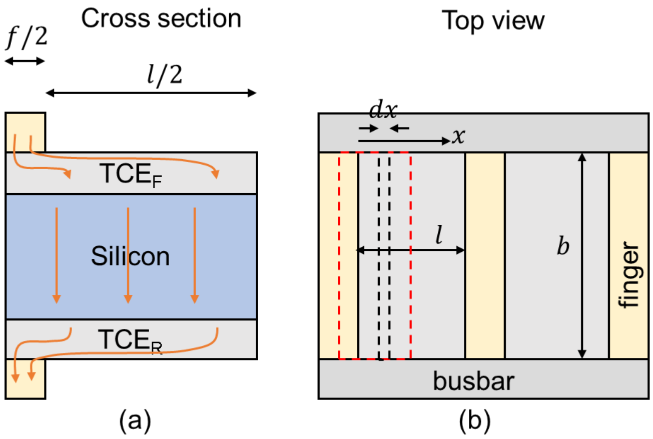

Figure S10. Unit domain of a silicon heterojunction solar cell with d representing the finger width, and l the finger pitch. a. Lateral view of two-terminal tandem with front and rear-TCE for bifacial configuration. b. Top view of a two-terminal tandem, with *w* representing the cell width and *dx* an infinitesimal change in the *x* direction (perpendicular to the cell width *w*).

The lateral current flow I through the TCE is maximum at the midpoint between two fingers, $x = l/2$, and decreases linearly to zero value just underneath the contact finger where only contact resistivity contributes to resistance, but there is no lateral transport. It is calculated as:

$$I = J.b.x$$

Where J is the current density of the area of interest in the device, labelled with a red dotted-line box in Figure S6.b, and equivalent to an area = $w.l/2$:
The total power loss can be found by integrating the incremental resistance losses from $x = 0$ to $x = l/2$.

$$P_{loss} = \int I^2 dR = \int_0^{\frac{l}{2}} (J.b.x)^2 \, R_{sheet}^{TCE} . \frac{dx}{b}$$

$$P_{loss} = \frac{l^3}{24} J^2 b R_{sheet}^{TCE} = \frac{l^3}{24} \left(\frac{I}{b.l/2} \right)^2 b R_{sheet}^{TCE} = \frac{I^2 l}{6b} R_{sheet}^{TCE} = I^2 \left(\frac{R_{sheet}^{TCE} l}{6b} \right).$$

The effective series resistance loss contributed by the TCE resistivity is hence:

$$R_{s0} = \frac{R_{sheet}^{TCE} l}{6b}$$

The TCE is present at the front and rear and hence it will contribute twice as much series resistance:

$$R_s = 2 \frac{R_{sheet}^{TCE} l}{6b}$$

Following Anand's procedure [3], it is possible to lump the R_s originating from the TCE to the ideal diode model describing the operation of the entire solar cell, and use a figure of merit that accounts for the operation of the TCE in the target device.

In our case, we also use the formalism proposed by Black and McDonald [4], where a pseudo I-V curve can be drawn (implied open-circuit voltage vs current density iV_{OC} vs iJ), which accounts for transport, intrinsic and extrinsic recombination losses as:

$$iV_{OC} = \frac{kT}{q} \ln \left(\frac{\Delta n \, (N + \Delta n)}{n_{i,eff}} \right)$$

$$iJ = J_{gen} - \frac{qW\Delta n}{\tau_{eff}(\Delta n)}$$

$$\tau_{eff} = \frac{1}{\frac{1}{\tau_{int}} + J_0 \frac{\Delta n\,(N + \Delta n)}{W.q.n_{i,eff}^2}}$$

where k is the Boltzmann constant, T is temperature, q is the electron charge, Δn is the excess carrier concentration, N is the dopant concentration, and ni,eff is the effective intrinsic carrier concentration (including bandgap narrowing), τ_{eff} is the effective lifetime, and J_{gen} is the generation current. By varying Δn, it is possible to generate an implied curve of current density vs voltage, and find the maximum power point.

Here we follow Black's calculation, using a thickness W=100 µm and a Jgen_max = 43.47 mA/cm2, which changes under different thicknesses of the TCE as described below. The thickness corresponds to the intrinsic efficiency limit of an n-type Si cell obtained by Niewelt et al. [5].

The intrinsic carrier concentration including bandgap narrowing ni,eff = 9.65×10^9 cm^{-3} and the intrinsic lifetime τint are calculated following the parameterisation and models used by Niewelt et al. [5].

We set the J_0 parameter to the lowest reported on LONGI's world-record solar cells [6]. J_0=0.5 fA/cm^2.

Using the single-diode system as illustrated in **Figure S10**, we reduce the pseudo-efficiency obtained from Black's formalism by adding the effect of the TCE series resistance as:

$$V_{Cell} = iV_{oc} - R_s I = \; iV_{oc} - R_s\left(iJ.b.\frac{l}{2}\right)$$

$$V_{Cell} = iV_{oc} - iJ\,\frac{R_{sheet}^{TCE}\,l^2}{6}$$

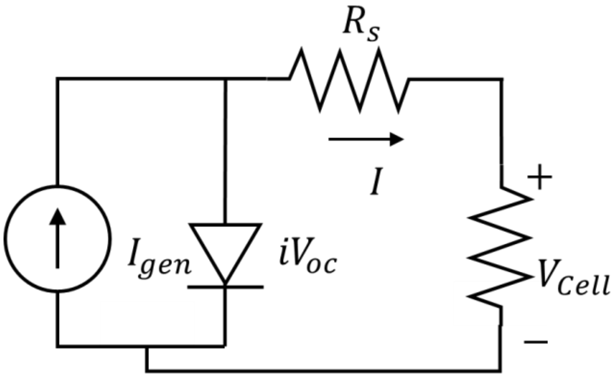

Figure S11. Ideal diode model of an ideal silicon solar cell only with series resistance losses.

The solutions are calculated for a range of V_{Cell} and the point of maximum power (V_m, I_m) is found to maximise $P_{max} = V_m * I_m$, in a Python algorithm, including in the open data archive deposit.

The interplay between generation current and finger pitch (l) is accounted for by executing a subroutine that calculates the optimal l based on the resistance contribution from the TCE versus the loss in J_sc from increased shading in small pitch distances. The generation current is scaled by the metallised faction:

$$J'_{gen} = J_{gen}(1 - f/l)$$

With the finger width set to 20 mm, as one of the smallest values reported in the literature for industrial screen printed contacts.

And the optimal l is found for each TCE sheet resistance. An example of the variation in efficiency as a function of finger spacing is given in **Figure S11**.

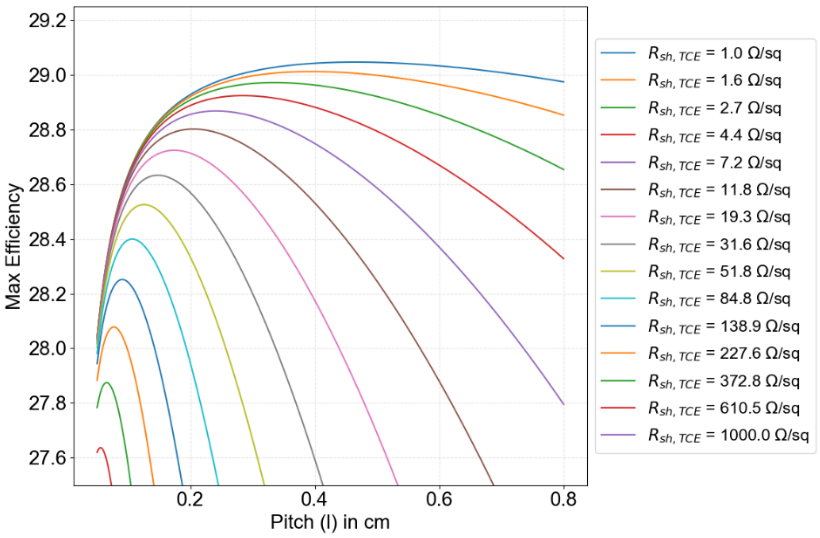

Figure S12. Variation in efficiency as a function of finger spacing.

Modelling the Generation current from the n,k optical properties of TCEs:

The generation current in 100 mm Si wafer was calculated using OPAL2 [7] as implemented by pvlighthouse.com.au. OPAL2 is a freeware program that rapidly simulates the optical performance of silicon solar cells by calculating optical losses such as reflection, absorption, and transmission across different angles of incidence. It achieves this through efficient ray tracing, which decouples from the Fresnel equations, minimising computation time. OPAL2 was used to model surface texture with random upright pyramids of characteristic angle 54.75°, using an AM1.5g spectrum normal to the surface, and a light trapping model where a factor Z is included in the absorption equation to represent the optical pathlength enhancement factor as:

$$A = 1 - e^{-\alpha Z W}$$

where *A* is the absorption, *α* is the absorption coefficient of the substrate, and *W* is the thickness of the substrate.

Z and A are selected [8] based on the scenario where (i) light inside the cell is isotropic, (ii) there is 100% internal reflection at the rear (or the cell is bifacially illuminated with equal intensity from both sides), and (iii) antireflection coatings are ideal, allowing 100% transmission for rays within the escape cone.
The Z and A models selected are

$$Z = 4 + \frac{\ln[n^2 + (1 - n^2)e^{-4\alpha W}]}{\alpha W}$$

$$A = \frac{1 - e^{-4\alpha W}}{1 - (1 - \frac{1}{n^2})e^{-4\alpha W}}$$

When varying the thicknesses of the TCEs, it is clear that the optical coupling changes from the change in interference inside the films. To make a comparison, all TCEs were capped with an antireflection coating (ARC) of MgF, and the thickness of the ARC was optimised to find the maximum value of generation current (J_{gen}). The three dielectric film system models are depicted in **Figure S12**. The absolute maximum possible is that of an optimal silicon oxide/nitride ARC.

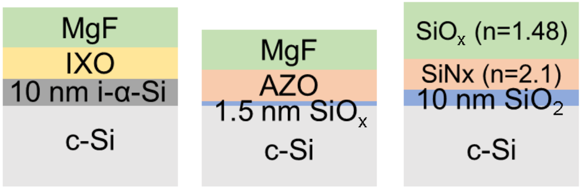

Figure S13. Three dielectric film system models.

References

1. Man, G., et al., *Electronically Passivated Hole-Blocking Titanium Dioxide/Silicon Heterojunction for Hybrid Silicon Photovoltaics.* Advanced Materials Interfaces, 2016. **3**(15).
2. www.pvlighthouse.com.au, *PV LightHouse*. 2022. p. Cells calculators-Cells calculators.
3. Anand, A., et al., *Introduction of a Novel Figure of Merit for the Assessment of Transparent Conductive Electrodes in Photovoltaics: Exact and Approximate Form.* Advanced Energy Materials, 2021. **11**(26).
4. Black, L.E. and D.H. Macdonald, *Improved Auger recombination models: Consequences for c-Si solar cells.* Solar Energy Materials and Solar Cells, 2022. **246**.
5. Niewelt, T., et al., *Reassessment of the intrinsic bulk recombination in crystalline silicon.* Solar Energy Materials and Solar Cells, 2022. **235**.
6. Lin, H., et al., *Silicon heterojunction solar cells with up to 26.81% efficiency achieved by electrically optimized nanocrystalline-silicon hole contact layers.* Nature Energy, 2023. **8**(8): p. 789-799.
7. McIntosh, K.R. and S.C. Baker-Finch, *OPAL 2: Rapid Optical Simulation of Silicon Solar Cells.* Proceedings of the 38th IEEE Photovoltaic Specialists Conference, Austin, in-press, 2012.
8. Green, M.A., *Lambertian light trapping in textured solar cells and light-emitting diodes: analytical solutions.* Progress in Photovoltaics: Research and Applications, 2002. **10**(4): p. 235-241.
